# Rare Clinical Sign of Hodgkin’s Lymphoma: Ocular Involvement

**DOI:** 10.4274/tjo.92609

**Published:** 2017-04-01

**Authors:** Ziya Ayhan, Revan Yıldırım Karabağ, İnci Alacacıoğlu, Süleyman Kaynak, Nilüfer Koçak

**Affiliations:** 1 Dokuz Eylül University Faculty of Medicine, Department of Ophthalmology, İzmir, Turkey; 2 Manisa State Hospital, Ophthalmology Clinic, Manisa, Turkey; 3 Dokuz Eylül University Faculty of Medicine, Department of Hematology, İzmir, Turkey

**Keywords:** Anterior uveitis, Hodgkin’s lymphoma, Macular edema, posterior uveitis

## Abstract

Bilateral non-granulomatous anterior uveitis with left vitritis and macular edema were detected in a 19-year-old woman presenting with blurred vision in her left eye. Light microscopic study of the pathologic mediastinal lymph node that was detected via contrast computed tomography imaging during etiologic study revealed nodular sclerosing and mixed cellularity Hodgkin’s lymphoma (HL). Ocular findings completely resolved with adriablastin, bleomycin, vinblastine, dacarbazine chemotherapy treatment. Herein, it is emphasized that HL should be remembered as one of the differential diagnoses in patients with ocular inflammatory pathologies such as uveitis and vasculitis. The ocular findings of HL are discussed.

## INTRODUCTION

Hodgkin’s lymphoma (HL) is a disease originating from lymphoid tissues and accounts for less than 1% of all cancers.^1^ As lymph nodes are distributed throughout the body, lymphomas may manifest with involvement in various areas.^2^ This can cause difficulties in diagnosis as well as delayed treatment.

Ocular involvement is more prevalent in non-HL compared to HL.^3^ There are many reports in the literature of ocular involvement in HL, which is reported to generally develop after HL diagnosis.^4,5,6^ Though rare, there are also patients who present with ocular symptoms and are subsequently diagnosed with HL.^7,8^

With this case report, we aimed to discuss the case of a 19-year-old patient who presented with complaints of low vision and was diagnosed with HL after testing, to examine the relationship between ocular findings and HL within the context of the literature, and to raise awareness of this condition.

## CASE REPORT

**Figure 1:**
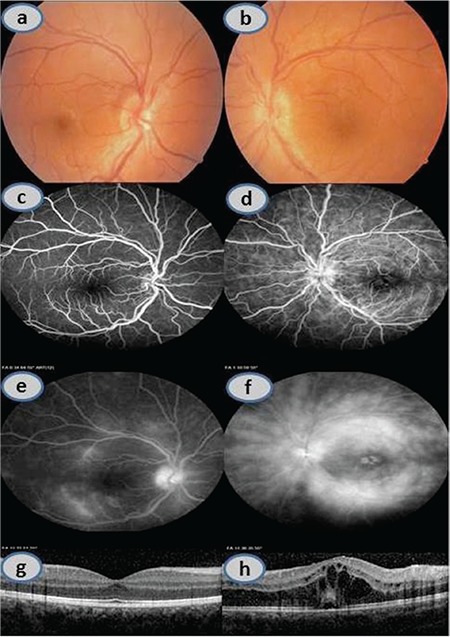
Color fundus photography from January 2012 showing normal OD (a) and indistinct optic disc margins and macular edema in OS (b). In early phase fluorescein angiography, OD appears normal (c) and leakage is evident in the disc and macula of the OS (d). In late phase, there is pronounced leakage around the disc and arcades in OD (e) and extensive leakage around the disc and arcades with macular edema in OS (f). Optical coherence tomography shows normal appearance in OD (g) and macular edema in OS (h) OD: Right eye, OS: Left eye

A 19-year-old female patient presented to our clinic with complaints of reduced vision in her left eye. The patient reported that her symptoms of reduced vision in the left eye had started 6 months earlier and that she had sought treatment for the first time 1 week earlier at a private ophthalmology outpatient clinic, from which she was referred to our clinic. On ophthalmologic examination, her best corrected visual acuity (BCVA) was 10/10 (0.0 logMAR) in the right eye and 2/10 (0.7 logMAR) in the left eye. Slit-lamp examination revealed bilateral non-granulomatous keratic precipitates and 2+ flare which were more pronounced in the left eye. Fundus examination was normal in the right eye, while macular edema and +/++ vitritis were observed in the left eye, though there was no vitreous turbidity (Figure 1a and 1b). Fluorescein angiography revealed extensive leakage from the vascular arcades in both optic discs and macular edema in the left eye (Figure 1c, 1d, 1e, and 1f). On optic coherence tomography (OCT), the right macula appeared normal, and severe cystoid macular edema was observed in the left eye (Figure 1g and 1h). The patient had no known systemic disease, but it was learned that she had lost 10 kg in the last 6 months using an herbal drug, that she tired quickly, and had complaints of numbness in the soles of her feet for the past 1.5 months. Topical treatment was initiated with 1% prednisolone acetate every hour and 1% cyclopentolate HCl 3 times daily. The patient was admitted to our clinic for etiologic study and treatment. Dermatologic examination and tests performed for differential diagnosis of Behçet’s disease were normal; neurologic examination and tests revealed no pathologies other than Chiari malformation observed on brain magnetic resonance imaging (MRI). Diffusion brain MRI and orbital MRI were normal. Electromyography test revealed no pathology other than mild sensory neuropathy. Except for elevated sedimentation rate (48 mm/hour) and leukocytosis, no pathology was detected in rheumatologic examination and immunologic tests. Following consultation with the department of pulmonary and respiratory diseases to establish etiology, contrast chest computed tomography revealed mediastinal lymph nodes of pathologic size. Hematology evaluated the condition as lymphoproliferative disease and recommended excisional lymph node biopsy. Atypical cells were found in the peripheral smear. The excised mediastinal lymph node was determined by pathologic examination to be consistent with classic nodular sclerosis and mixed cellularity HL. The patient was diagnosed with stage 3B HL and transferred to the hematology department for advanced tests and treatment with 6 courses of adriablastin, bleomycin, vinblastine, and dacarbazine (ABVD).

**Figure 2:**
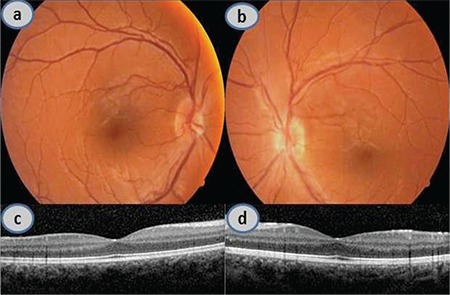
Appearance is normal in color fundus photograph (a, b) and optical coherence tomography images (c, d) from April 2012

In ophthalmologic examination conducted in April 2012, after 6 courses of chemotherapy, the patient’s BCVA was 10/10 (0.0 logMAR) in both eyes. Slit-lamp examination revealed complete resolution of the anterior uveitis findings. The optic disc, macula, and peripheral retina of both eyes appeared normal in fundus examination. OCT revealed complete regression of the macular edema in the left eye (Figure 2a, 2b, 2c and 2d). Furthermore, her HL was in full remission after chemotherapy. No recurrence of ocular symptoms or HL was observed in follow-up examinations through February 2015.

## DISCUSSION

HL is usually seen in individuals aged 15-34 years and those over 55 years old.1 The incidence of pediatric HL tends to rise as family size increases and socioeconomic status decreases; the opposite has been reported with the adult form, which is associated with high socioeconomic status in industrialized nations.^9^ Although HL is more prevalent among males in all age groups, the nodular sclerosis subtype is more common among females.^9^ Unlike most other cancers, HL can be cured, generally through a combination of radiotherapy and chemotherapy.^10^ Intraocular involvement is rare in lymphomas, and diagnosis may be delayed due to its nonspecific signs and possible masking by inflammatory processes. For this reason, HL should be included in the differential diagnosis of ocular inflammatory pathologies such as uveitis and vasculitis.

Ocular involvement in HL occurs by various mechanisms including direct lymphomatous or metastatic involvement of the choroid and retina; paraneoplastic vasculitis; and iatrogenic complications arising from HL treatment or immunosuppression.^5,6,7,11^ These patients may exhibit infiltration of the ocular structures, retinal periphlebitis, focal chorioretinitis, vitritis, papillary edema, exudative retinal detachment, soft exudates, retinal hemorrhage, necrotizing retinitis, peripheral retinal exudates, and retinal white spots.^3,12^ In a study by Towler et al.^8^ comparing 2 patients whose posterior uveitis preceded HL diagnosis and 2 patients who presented with posterior uveitis after HL diagnosis, the authors reported that the two groups showed no differences in ocular lesion type. Mateo-Montoya et al.^12^ reported a patient with macular edema, papillitis, and retinal white spots in the posterior pole and equatorial region who was diagnosed with HL after systemic etiologic study; they noted that the patient’s ocular signs completely resolved after chemotherapy and radiotherapy. In our case, both non-granulomatous anterior uveitis and vitritis with macular edema were detected as the first clinical signs of HL.

Granulomatous inflammation is a known sign of HL.^8^ The exact source of this granulomatous reaction is unknown, though it is thought to possibly result from an immune reaction to tissue response an underlying viral infection. It has been reported that Epstein-Barr virus (EBV) latent gene products affect lymphomatogenesis and are associated with aggressive subtypes of HL.^13^ However, nodular sclerosis HL, the subtype found in our patient, was found to be the subtype least associated with EBV latent membrane protein.^14^ Moreover, it has been reported that the anterior uveitis seen in HL, which is composed of macrophages, epithelioid cells, and lymphocytes, may be related to this granulomatous reaction. Barkana et al.^15^ also detected atypical granulomatous keratoconjunctivitis in a 19-year-old patient and diagnosed HL based on an inguinal lymph node biopsy.

Towler et al.^8^ reported achieving complete remission of ocular inflammation after chemotherapy. They attributed this to chemotherapeutic suppression of the ocular inflammatory response, as well as the reduction of the inflammatory response to malignant cells due to their destruction. After 6 courses of ABVD therapy for stage 3B HL, our patient also showed full HL remission and complete regression of her ocular symptoms.

## CONCLUSION

In brief, this case highlights the fact that ophthalmologists should be vigilant for potential malignant lesions in patients with ocular signs like uveitis and vasculitis, that these may be indicators of a life-threatening systemic disease, and that systemic investigation is crucial for diagnosis and treatment.
